# Phospholipidome of endothelial cells shows a different adaptation response upon oxidative, glycative and lipoxidative stress

**DOI:** 10.1038/s41598-018-30695-0

**Published:** 2018-08-17

**Authors:** Simone Colombo, Tânia Melo, Marta Martínez-López, M. Jesús Carrasco, M. Rosário Domingues, Dolores Pérez-Sala, Pedro Domingues

**Affiliations:** 1 Mass Spectrometry Centre, Department of Chemistry & QOPNA, University of Aveiro, Campus Universitário de Santiago, 3810-193 Aveiro, Portugal; 2 Department of Structural and Chemical Biology, Centro de Investigaciones Biológicas, CSIC, Ramiro, de Maeztu, 9, 28040 Madrid, Spain

## Abstract

Endothelial dysfunction has been widely associated with oxidative stress, glucotoxicity and lipotoxicity and underlies the development of cardiovascular diseases (CVDs), atherosclerosis and diabetes. In such pathological conditions, lipids are emerging as mediators of signalling pathways evoking key cellular responses as expression of proinflammatory genes, proliferation and apoptosis. Hence, the assessment of lipid profiles in endothelial cells (EC) can provide valuable information on the molecular alterations underlying CVDs, atherosclerosis and diabetes. We performed a lipidomic approach based on hydrophilic interaction liquid chromatography-tandem mass spectrometry (HILIC-MS/MS) for the analysis of the phospholipidome of bovine aortic EC (BAEC) exposed to oxidative (H_2_O_2_), glycative (glucose), or lipoxidative (4-hydroxynonenal, HNE) stress. The phospholipid (PL) profile was evaluated for the classes PC, PE, PS, PG, PI, SM, LPC and CL. H_2_O_2_ induced a more acute adaptation of the PL profile than glucose or HNE. Unsaturated PL molecular species were up-regulated after 24 h incubation with H_2_O_2_, while an opposite trend was observed in glucose- and HNE-treated cells. This study compared, for the first time, the adaptation of the phospholipidome of BAEC upon different induced biochemical stresses. Although further biological studies will be necessary, our results unveil specific lipid signatures in response to characteristic types of stress.

## Introduction

The endothelium is the tissue responsible for the regulation of the hemodynamics of the whole circulatory system. Endothelial cells (EC) under oxidative stress play a pathogenic role in the onset and the development of cardiovascular diseases (CVDs)^1,2^ and atherosclerosis^3^. The scenario in which the endothelium is involved in the initiation and progression of CVDs and other oxidative stress-related disorders is referred to as endothelial dysfunction, an array of maladaptive changes in the functional phenotype of EC that is known to occur upon exposure to minimally oxidized low-density lipoproteins (LDL) and overproduction of reactive oxygen species (ROS)^2,4,5^. Moreover, diabetes and hyperglycemia can also trigger oxidative stress, leading to endothelial dysfunction, which contributes to diabetic retinopathy^6,7^, cardiovascular complications^8^ and atherosclerosis^9^. Also, hyperglycemia has been associated with endothelial dysfunction through the decrease of cell viability and the induction of EC apoptosis^9,10^. Oxidative stress can also lead to the formation of aldehydic lipid peroxidation products (ALPP), as 4-hydroxy-2-nonenal (HNE), which can further induce oxidative stress in EC^11–13^. HNE is able to exert prominent cytotoxic effects in human umbilical EC (HUVEC) that result in morphological changes, diminished cellular viability, impaired endothelial barrier function, and cell apoptosis^11^. Therefore, the endothelial barrier dysfunction promoted by HNE may contribute to the vascular changes that lead to the development of atherosclerosis^11,14^.

**Figure 1:**
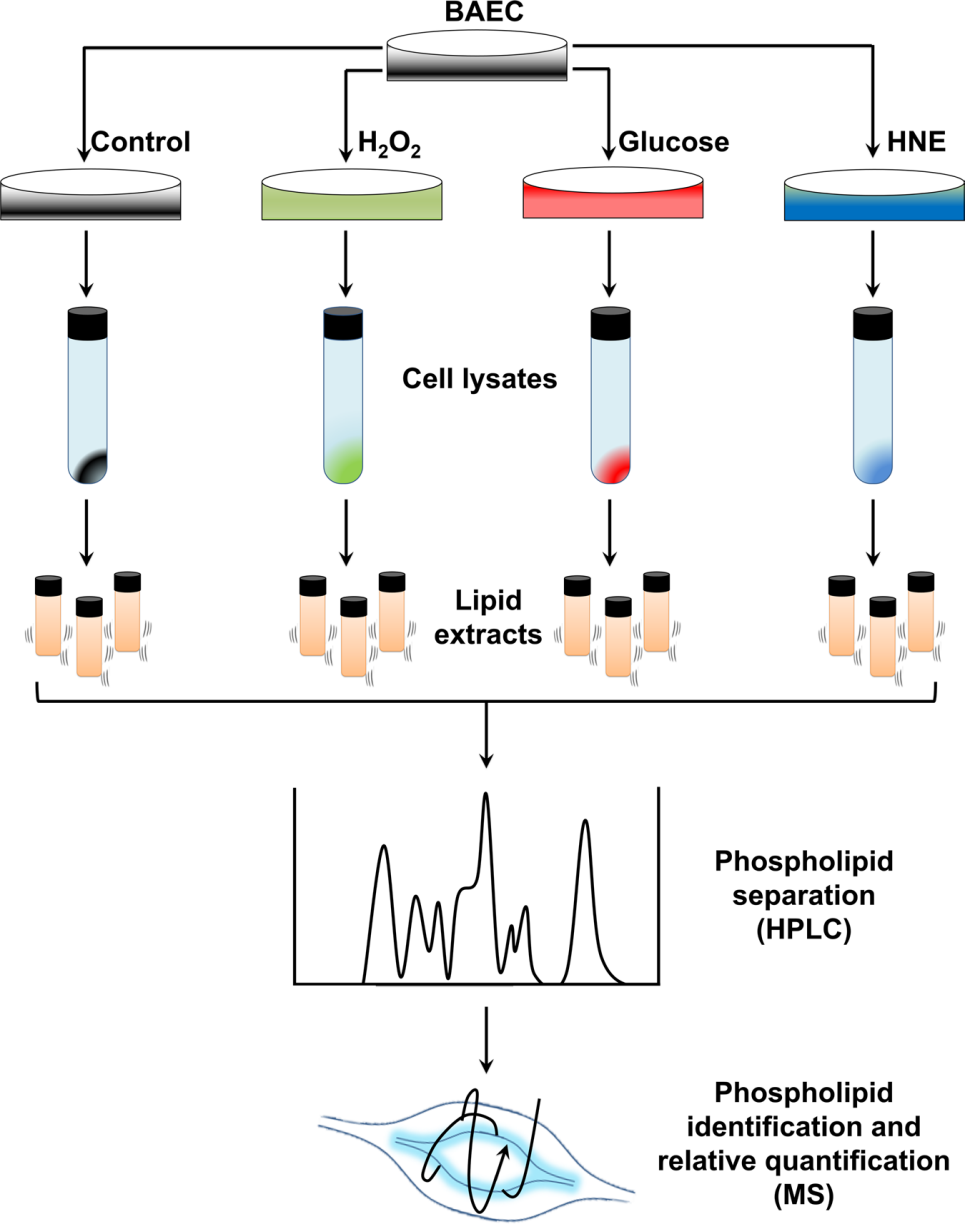
Schematic diagram showing the experimental workflow including cell treatment, cell lysis, lipid extraction, chromatographic separation and MS analysis.

In the CVDs that are related to endothelial dysfunction and, more broadly, in chronic inflammatory diseases related to oxidative stress, lipids have progressively been considered as key molecules mediating the outbreak and the progression of such pathologies^15–17^. During the last decade, we have assisted to the rapid development of lipidomics, a group of analytical platforms and protocols aimed at the assessment of lipid metabolic profiles and networks in biological systems^18^. Lipidomics can provide information about the molecular basis of CVDs, highlight the links between lipid functions and pharmacological treatments, and allow a more in-depth monitoring of the response to therapies^19^. However, the evaluation of the lipidome of EC is still limited. Murphy and co-authors^20^ reported the phospholipid (PL) compositions of cultured EC from human artery, saphenous vein, and umbilical vein, and observed a similar profile for the three cell types. Héliè-Toussaint and co-authors^21^ further studied the lipidomic pathways of HUVEC, observing a preferential homeostasis leading to the synthesis of PL rather than triacylglycerols, and a fast incorporation of palmitic acid and arachidonic acid in the membrane PL pool. More recent insights in cardiovascular lipidomics have allowed the characterization of the lipidome of human atherosclerotic plaques, pinpointing an enrichment in phosphatidylcholines (PC), oxidized phosphatidylcholines (ox-PC) and lyso-PL within the cells^22,23^. Nevertheless, the understanding of the pathogenic mechanisms underlying CVDs requires the study of the phospholipidome of EC upon stressing conditions such as hyperglycemia and overproduction of ROS. However, up to date, only Yang and co-authors^24^ have investigated the variations in the lipidome of human EC upon oxidative stress. A phospholipidomic fingerprinting of EC subjected to biochemical stress would represent a very informative model of cardiovascular pathobiology, aimed to understand the molecular mechanisms of adaptation that may occur during endothelial dysfunction and contribute to the onset of CVDs. In the present study, we wanted to assess whether specific stress conditions would induce distinctive changes in the lipidome of EC. For this, we employed hydrophilic interaction liquid chromatography coupled to mass spectrometry (HILIC-MS/MS) for the phospholipidomic profiling of cultured bovine aortic EC (BAEC), which constitute a widely used model for vascular biology studies, upon oxidative (H_2_O_2_), glycative (glucose) or lipoxidative (HNE) stress conditions. The workflow used to carry out the entire experiment is shown in Fig. 1. Our results show for the first time that the lipidome of EC is exquisitely responsive to diverse stress conditions, and thus, may mediate specific adaptive changes.

## Results

Lipids have recently emerged as key mediators in the onset of chronic inflammatory diseases characterized by endothelial dysfunction and oxidative stress. Hence, we employed a HILIC-LC-MS/MS platform to analyse the phospholipid profile of BAEC treated in control conditions and in response to different stressing agents (H_2_O_2_, glucose, or HNE). The data sets resulting from the HILIC-LC-MS/MS analysis of four sample groups (control, H_2_O_2_, glucose, and HNE) were later subjected to both univariate and multivariate statistical analysis, aiming to identify significant changes occurring in the BAEC phospholipid profile upon induced biochemical stress.

**Figure 2:**
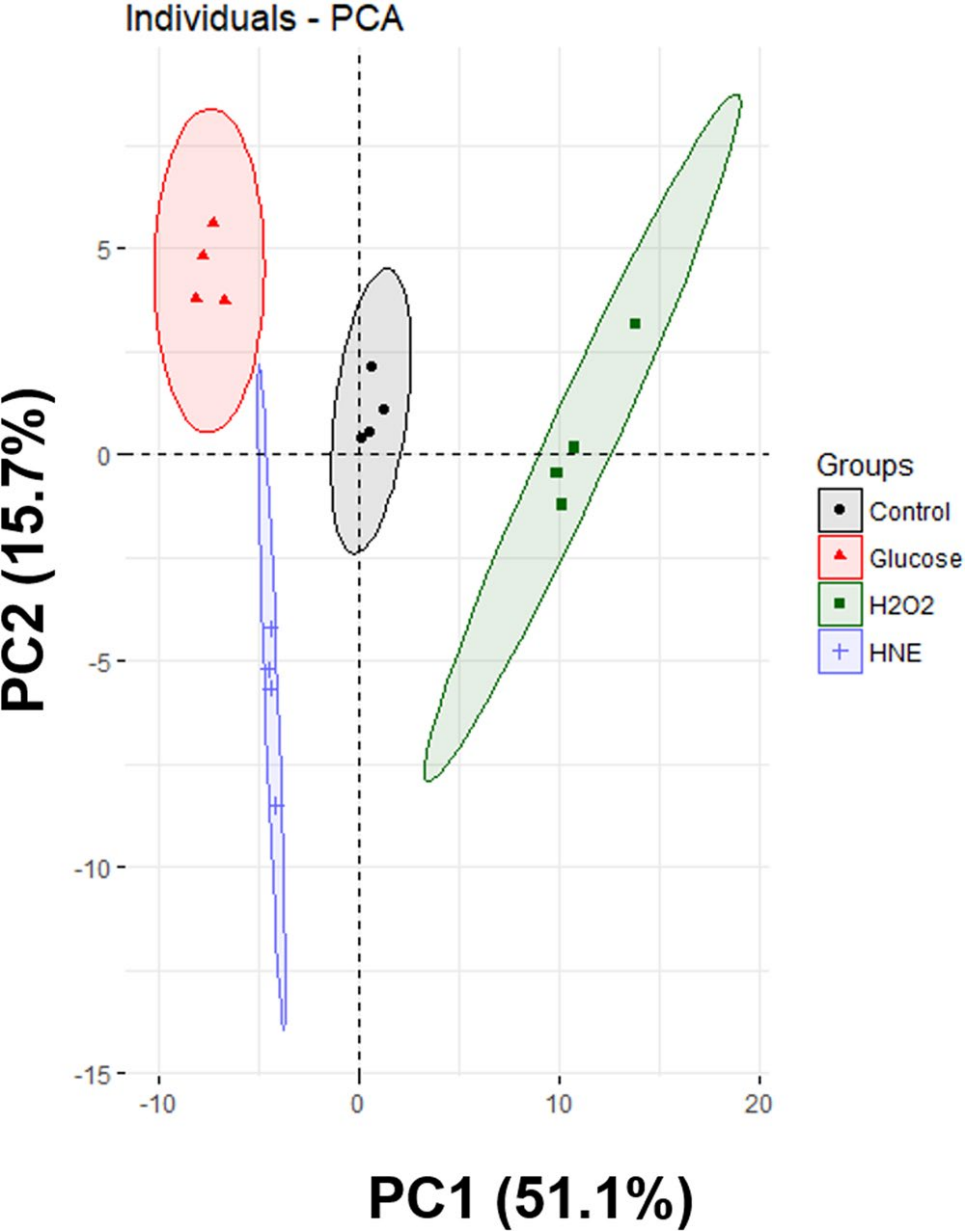
Principal component analysis score plot of the phospholipid profiles obtained from BAEC treated with glucose, H_2_O_2_ or HNE. Control, vehicle medium; Glucose, 25 mM glucose; H_2_O_2_, 1 mM hydrogen peroxide; HNE, 10 µM 4-hydroxy-2-nonenal. All samples were analysed after 24 hours of treatment.

We performed the identification and the relative quantification of PL species belonging to 8 different classes: phosphatidylcholine (PC), phosphatidylethanolamine (PE), phosphatidylserine (PS), phosphatidylglycerol (PG), phosphatidylinositol (PI), lyso-PC (LPC), cardiolipin (CL) and sphingomyelin (SM). The whole list of the 109 PL species (correspondent to the most abundant species in all the identified classes) that were identified and quantified after MS and MS/MS analysis of each sample can be found in Supplementary Table S1. The total chain length (C) and degree of unsaturation (N) are included. Also, the different isomers of the same classes that bear different esterified fatty acids and correspond to each C:N composition were included. These isomers cannot be resolved by the LC-HILIC method, but the fatty acyl composition was determined by MS/MS analysis. Negative ion mode MS/MS data were used to analyse fatty acid carboxylate anions fragments, which allowed to assign the fatty acyl chains esterified to the PL molecular species. For the relative quantification of all the PL listed in Supplementary Table S1, the peak areas of the extracted ion chromatograms (XICs) of each PL species (C:N) within each class were normalized using the peak area of the internal standard (IS) selected for the class. Data were subsequently autoscaled and then subjected to a principal component analysis (PCA) to display the clustering trends of the four experimental groups of BAEC: control, H_2_O_2_-, glucose-, and HNE-treated. The PCA showed that all the groups were separated from each other in a two-dimensional score plot which represented the analyses describing 66.8% of the total variance, including principal component 1 (51.1%) and principal component 2 (15.7%), where principal component 1 was the major discriminating component (Fig. 2). From the loading values, PE (34:3), PE (36:2), PE (38:2), PE (36:1) and PE (36:3) were the major contributors from component 1, whereas PI (36:2), PE (34:1), PC (O-32:0), PI (36:1) and PG (34:2) were the main contributors for component 2. Control samples were scattered on the central region of the plot. Glucose or HNE-treated samples were scattered on the left region of the plot according to the order: glucose, HNE. Interestingly, H_2_O_2_-treated samples formed the only group that was scattered on the right region of the plot.

**Figure 3:**
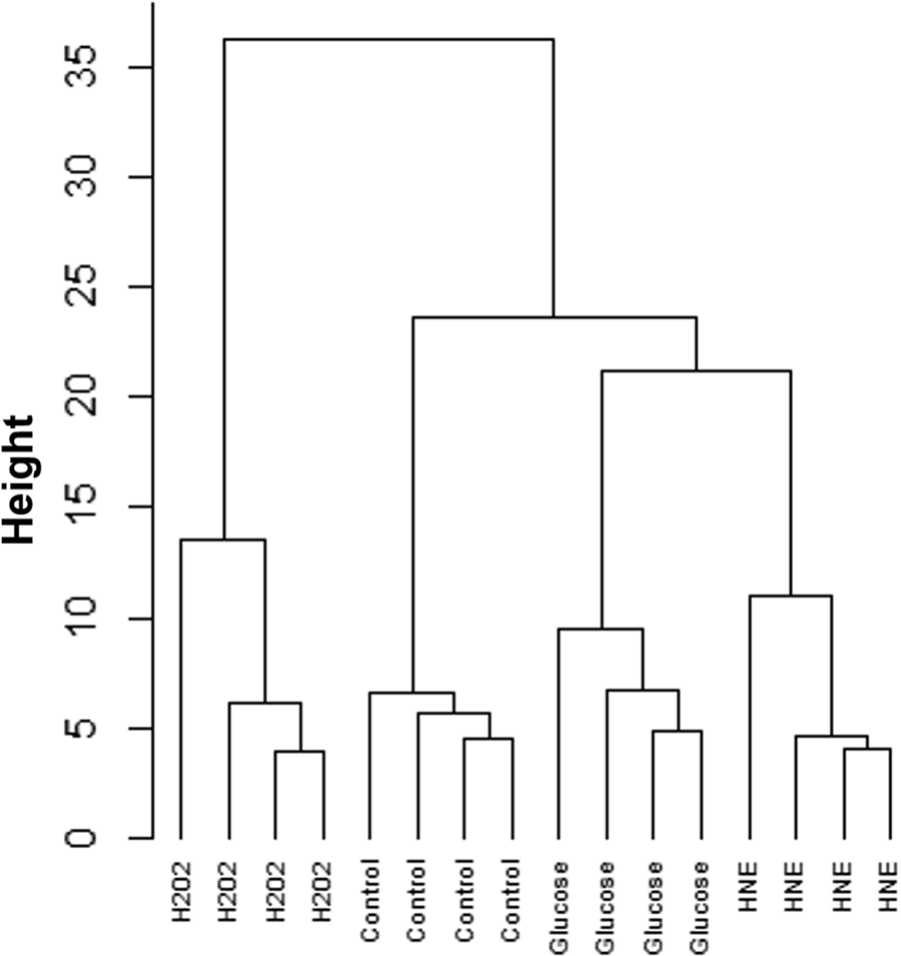
Hierarchical cluster analysis of the phospholipid profiles obtained from BAEC treated with glucose, H_2_O_2_ or HNE. Control, vehicle medium; Glucose, 25 mM glucose; H_2_O_2_, 1 mM hydrogen peroxide; HNE, 10 µM 4-hydroxy-2-nonenal. All samples were analysed after 24 hours of treatment.

Additionally, we carried out a hierarchical clustering analysis (HCA) on the phospholipid data sets from the four conditions (Fig. 3). The resulting HCA dendrogram depicted a noticeable separation of the four data sets of control, H_2_O_2_, glucose, and HNE. The first level of separation was evidenced between H_2_O_2_-treated samples and the remaining conditions. The second level of separation distinguished control samples from the two remaining groups (glucose and HNE-treated). However, glucose- and HNE-treated cells differentiated in two clusters in the third level of separation.

**Figure 4:**
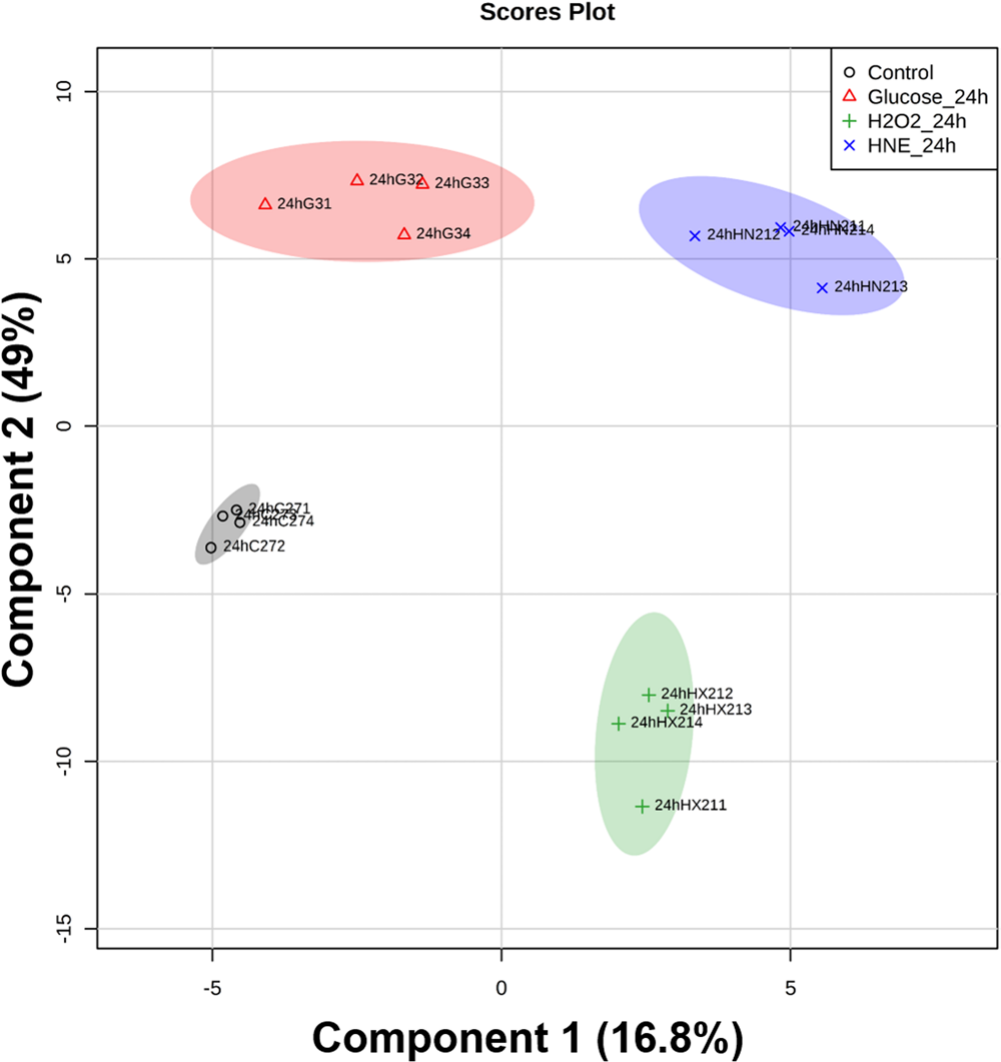
Projection to latent structures discriminant analysis score plot of the phospholipid profiles obtained from BAEC treated with glucose, H_2_O_2_ or HNE. Control, vehicle medium; Glucose, 25 mM glucose; H_2_O_2_, 1 mM hydrogen peroxide; HNE, 10 µM 4-hydroxy-2-nonenal. All samples were analysed after 24 hours of treatment.

Then, we performed a projection to latent structures discriminant analysis (PLS-DA) in order to maximize the phenotypic classification of samples, which showed the performance statistics of R2X = 0.94241, R2Y = 0.96805 and a high prediction parameter Q2 of 0.80026 (X) and 0.90986 (Y). The four groups were well separated in the resulting two-dimensional score plot (Fig. 4). The PLS-DA score plot described 65.8% of the total variance, including component 1 (16.8%) and component 2 (49%). Along with component 2, control samples were scattered at the central region of the plot. Glucose- and HNE-treated samples were scattered on the top region of the plot. H_2_O_2_-treated samples formed the only group that was scattered at the bottom region of the plot. Along with component 1, control- and glucose-treated samples were scattered at the left side of the plot, while HNE- and H_2_O_2_-treated samples were scattered on the right side of the plot.

Besides multivariate statistical analyses, we used another approach to facilitate the interpretation of the extensive dataset produced in the present study, namely the analysis of semi-quantitative phospholipidomic features across distinct depths of detail.

**Figure 5:**
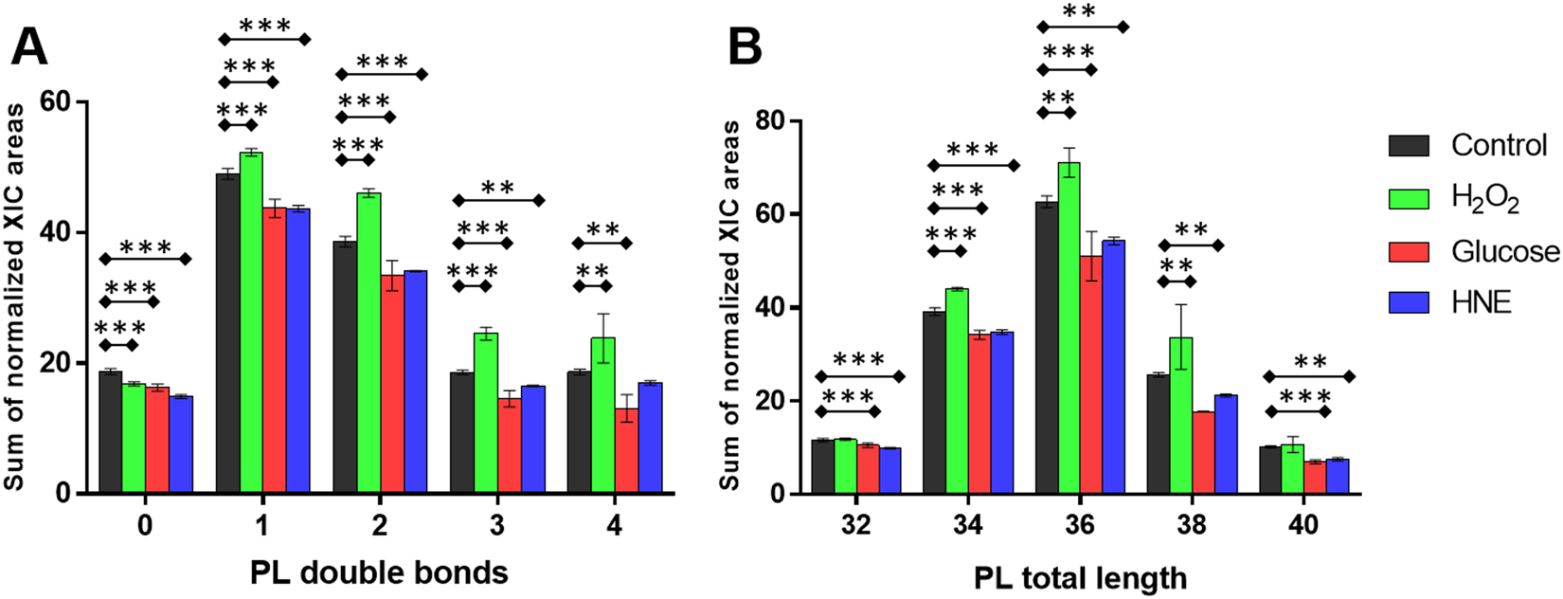
(**A**) Phospholipid species comprised of the same number of unsaturation on the fatty acyl chains, given as normalized XIC area for each category (the contribution of CL is not included for clarity). (**B**) Phospholipid species comprised of the same number of carbon atoms on the hydrocarbon chains, given as normalized XIC area for each category (the contributions of CL and LPC are not included for clarity). *^,^**^,^*** Statistically significant variation between selected conditions (p < 0.05, p < 0.01 and p < 0.001, respectively).

For a clearer overview of the phospholipidome, we included the analysis of PL by features, assessing the adaptation of all the PL species bearing the same number of unsaturation, or bearing the same total carbon chain length, as already performed by other authors^25^. Therefore, the cumulative levels of all the PL species comprised of the same number of unsaturation, ranging from zero, one, two, three and four double bonds, were summarized in the singular lipidomic features PL-DB0, PL-DB1, PL-DB2, PL-DB3, and PL-DB4, respectively. When comparing globally the degree of unsaturation observed for BAEC PL upon the four tested conditions (control, H_2_O_2_, glucose and HNE), the highest cumulative levels of (poly-)unsaturated species (PL-DB1, PL-DB2, PL-DB3, and PL-DB4) were always observed after the treatment with H_2_O_2_ (Fig. 5). Conversely, treatment of BAEC with glucose and HNE downregulated the levels of PL-DB1, PL-DB2, PL-DB3 and PL-DB4 when compared with control (Fig. 5). Only when comparing HNE with control, changes in PL-DB4 were not statistically significant.

Analogously, the cumulative levels of all the PL species comprised of the same number of carbons in their hydrocarbon chain were summarized in the singular lipidomic features PL-C32, PL-C34, PL-C36, PL-C38, and PL-C40, respectively. Regardless of the treatment, PL-C36 species were always the most abundant in BAEC. Treatment of BAEC with H_2_O_2_ lead to an increase of the features PL-C34, PL-C36, and PL-C38 when compared with control (Fig. 5). Conversely, we observed a downregulation of PL-C32, PL-C34, PL-C36, PL-C38, and PL-C40 for BAEC treated with glucose, and a downregulation PL-C32, PL-C34, PL-C36, and PL-C40 for BAEC treated with HNE, in comparison with control (Fig. 5). We did not observe any statistically significant variation for PL-C32 and PL-C40 after treatment with H_2_O_2_, nor for PL-C38 after treatment with HNE.

**Figure 6:**
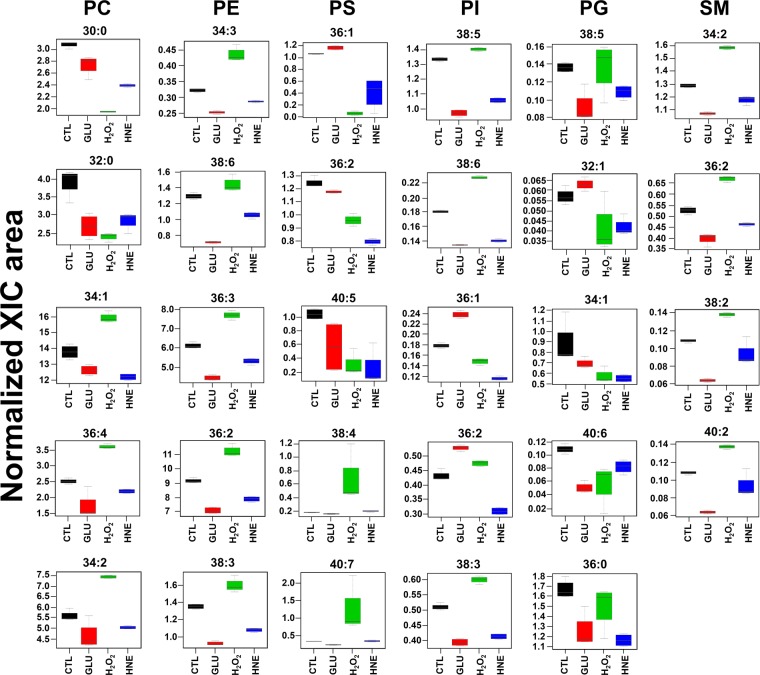
Box plots of the 24 most discriminant PL molecular species from BAEC treated with glucose, H_2_O_2_ or HNE. CTL, vehicle medium; GLU, 25 mM glucose; H_2_O_2_, 1 mM hydrogen peroxide; HNE, 10 µM 4-hydroxy-2-nonenal. All samples were analysed after 24 hours of treatment.

For a more detailed interpretation of the data, we further addressed the adaptation of single PL species induced by the different stressing treatments. The most abundant PC molecular species was PC (34:1) followed by PC (36:2), in all the conditions. We observed a statistically significant increase of the levels of PC (34:1), PC (34:2) and PC (36:4) in cells treated with H_2_O_2_, when compared to control cells (p < 0.05). On the other hand, we observed a statistically significant decrease of the levels of PC (30:0) and PC (32:0) in H_2_O_2_-treated cells in comparison with control. Interestingly, all these five PC molecular species, PC (34:1), PC (34:2), PC (36:4), PC (30:0) and PC (32:0), were decreased in high glucose-treated cells when compared to control cells (p < 0.05). PC (30:0), PC (32:0) and PC (34:1) were also decreased in cells treated with HNE in comparison with controls (p < 0.05). No significant alterations between HNE-treated cells and control cells were observed for PC (34:2) and PC (36:4) (Fig. 6 and Table 1).

**Table 1:** Summary of the alterations observed in the molecular species of PC, PE, PG, PS, PI, and SM from BAEC comparing control with H_2_O_2_-treated, control with glucose-treated and control with HNE-treated cells, along with their respective fold changes.

Class	Species (C:N)	H_2_O_2_ vs. control		Glucose vs. control		HNE vs. control	
		Adaptation	Fold change	Adaptation	Fold change	Adaptation	Fold change
PC	30:0	↓	1.60	↓	1.13	↓	1.30
	32:0	↓	1.64	↓	1.47	↓	1.38
	34:1	↑	0.87	↓	1.09	↓	1.13
	36:4	↑	0.70	↓	1.44		
	34:2	↑	0.75	↓	1.21		
PE	34:3	↑	0.73	↓	1.21	↓	1.13
	38:6	↑	0.90	↓	1.79	↓	1.22
	36:3	↑	0.79	↓	1.37	↓	1.15
	36.2	↑	0.82	↓	1.29	↓	1.16
	38:3	↑	0.84	↓	1.48	↓	1.26
PG	38:5			↓	1.49	↓	1.25
	32:1	↓	1.40			↓	1.36
	34:1	↓	1.53	↓	1.27	↓	1.59
	40:6	↓	1.87	↓	2.14	↓	1.32
	36:0			↓	1.34	↓	1.44
PS	36:1			↓	0.91	↓	2.61
	36:2	↓	1.30	↓	1.06	↓	1.56
	40:5	↓	3.57	↓	1.85	↓	4.40
	38:4	↑	0.26				
	40:7	↑	0.29				
PI	38:5	↑	0.95	↓	1.38	↓	1.26
	38:6	↑	0.79	↓	1.36	↓	1.30
	36:1	↓	1.20	↑	0.75	↓	1.55
	36:2	↑	0.91	↑	0.82	↓	1.39
	38:3	↑	0.85	↓	1.29	↓	1.23
SM	34:2	↑	0.81	↓	1.21	↓	1.10
	36:2	↑	0.78	↓	1.32	↓	1.14
	38:2	↑	0.79	↓	1.70	↓	1.17
	40:2	↑	0.82	↓	1.36		

All the alterations are significant at the p < 0.05 level. CTL, vehicle medium; GLU, 25 mM glucose; H_2_O_2_, 1 mM hydrogen peroxide; HNE, 10 µM 4-hydroxy-2-nonenal. All samples were analysed after 24 hours of treatment.

In all the conditions, PE (36:2) was the most abundant PE molecular species followed by PE (36:3). The levels of PE (36:3), along with PE (34:3), PE (38:6), PE (36:2) and PE (38:3), were increased in H_2_O_2_-treated cells when compared to controls (p < 0.05). Conversely, we observed a statistically significant down-regulation for all these five PE species in both glucose- and HNE-treated cells compared to controls (p < 0.05) (Fig. 6 and Table 1).

When comparing cells treated with H_2_O_2_ with controls we found that PS (38:5), PS (38:4) and PS (40:7) levels were increased, while PS (36:2) and PS (40:5) were decreased (p < 0.05). We also observed a significant decrease of PS (36:2), PS (40:5) and PS (36:1) in both glucose and HNE-treated cells compared with controls (p < 0.05) (Fig. 6 and Table 1).

A significant increase of the levels of PI (38:5), PI (38:6), PI (36:2) and PI (38:3), and a significant decrease of PI (36:1), were observed in cells treated with H_2_O_2_ compared to control cells (p < 0.05). When comparing glucose-treated cells with controls, the levels of PI (38:5), PI (38:6), PI (38:3) were decreased while PI (36:1) and PI (36:2) were increased (p < 0.05). We also observed a statistically significant decrease for all these five PI species in cells treated with HNE when compared with controls (p < 0.05) (Fig. 6 and Table 1).

The treatment of cells with H_2_O_2_ induced a decrease on the levels of PG (32:1), PG (34:1) and PG (40:6), compared to controls (p < 0.05). We also observed decrease of the levels of PG (34:1) and PG (40:6), along with PG (38:4) and PG (36:0), in glucose-treated cells when compared with controls. The levels of five PG molecular species - PG (32:1), PG (34:1), PG (40:6), PG (38:5) and PG (36:0) – were found to be decreased in cells treated with HNE compared to controls (p < 0.05) (Fig. 6 and Table 1).

The most abundant SM molecular species in all conditions was SM (34:1). We observed increased levels of the species SM (34:2), SM (36:2), SM (38:2), SM (40:2) in cells treated with H_2_O_2_, compared to controls (p < 0.05). Conversely, all these five species of SM were decreased in glucose-treated cells, when compared to controls (p < 0.05). Treatment of cells with HNE also decreased the levels of the species SM (34:2), SM (36:2), SM (38:2), in comparison with controls (Fig. 6 and Table 1).

We did not observe any statistically significant difference for the levels of the molecular species belonging to the CL and LPC classes.

## Discussion

Lipids have been highlighted as biomolecules involved in the onset of CVDs, a family of chronic inflammatory diseases in which vascular pathobiology is associated with endothelial dysfunction. Cellular lipid profiling can provide evidence at the molecular level for the role of lipids in these diseases. In our study, we performed a phospholipidomic profiling on BAEC subjected to H_2_O_2_, glucose, or HNE treatment, aiming to elucidate the adaptations in the PL of EC upon three models of biochemical stress associated with the onset and/or progression of CVDs.

In a first instance, the multivariate analyses that we performed (PCA, PLS-DA, and HCA) indicated that the phospholipid profiles of BAEC were significantly altered in response to each one of the induced biochemical stresses (H_2_O_2_, glucose or HNE). The two-dimensional plots of PCA (Fig. 2) and PLS-DA (Fig. 4), along with the dendrogram of HCA (Fig. 3), showed that the most evident differentiation of the BAEC-treated phospholipidome, in comparison with controls, occurred in the model of oxidative stress, 24 h after exposure to 1 mM H_2_O_2_. This suggests that the H_2_O_2_-mediated toxicity induced more particular and specific changes in the PL profile of BAEC when compared with glucotoxicity and lipotoxicity, presumably due to the adaptation mechanisms established by the cells to survive the exposure to oxidative stress. Yang and co-authors^24^ studied a hybrid cell line (EA.hy296) subjected to oxidative stress, and using phospholipidomic data found that samples were clearly clustered depending on the different exposure times to 0.2 mM H_2_O_2_ (0, 1, 2, 3 and 6 h).

Besides the present study, no other published works have reported the adaptation of the phospholipid profile of EC upon hyperglycemia. In our study, BAEC treated with 25 mM glucose for 24 h showed a significant alteration of the phospholipidome. PCA, HCA and PLS-DA (Figs 2, 3 and 4, respectively) all exhibited good clustering of the phospholipid profile of glucose-treated samples when compared to controls, differing from the trend observed for H_2_O_2_-treated samples. The clustering evidenced by HCA, in which high glucose samples are relatively closer to controls in comparison to H_2_O_2_ samples, suggests that the cellular effects mediated by glucose affected the phospholipid profile of BAEC via less harsh changes when compared to a directly induced acute oxidative stress.

In this study, we also assessed the phospholipidome of BAEC upon HNE exposure. Although the biological effects of HNE on several EC types have already been widely studied (as reviewed by Chapple and co-authors^12^), this is also the first study that aimed to evaluate the variations in the phospholipid profile of EC treated with an ALPP. In this study, we used 10 µM HNE, a concentration in the range of those measured in disease states^12^. BAEC treated for 24 h with HNE showed a significant phenotypical differentiation of their phospholipid profile, as shown in Figs 2, 3 and 4.

Treatment of BAEC with H_2_O_2_ increased the cumulative levels of (poly-)unsaturated PL species PL-DB1, PL-DB2, PL-DB3, and PL-DB4, in comparison with control (Fig. 5). Remarkably, it had been previously described that direct oxidative stress (H_2_O_2_ 1 mM) induced PL peroxidation within the first hours of treatment^26^, while along 24 hours of treatment, BAEC may have established an adaptive mechanism of re-acylation of unsaturated fatty acyl chains in the PL pool, aimed to counteract the ROS-mediated peroxidation. An increase in (poly)-unsaturated PL species was also reported by Peterson and co-authors^27^, in primary neurocortical cells incubated with H_2_O_2_ for 24 h. Hence, this is in agreement with our results that show augmented levels of (poly)-unsaturated species in H_2_O_2_-exposed BAEC. The amount of (poly)-unsaturated PL species in the plasma membrane is known to be an effective modulator of its physical properties, as phase transition temperature^28^. In our study, the up-regulation observed for the (poly)-unsaturated species of BAEC subjected to oxidative stress might have resulted in increased membrane fluidity. We found this adaptation of interest since an augmented membrane fluidity was also observed by Sergent^29^ in rat primary hepatocytes subjected to ethanol-induced oxidative stress, which would be by our conjectures. However, it is important to remark that in the present study the phospholipidome composition was not strictly studied for the plasma membrane. Hence PL (poly)-unsaturated species might also belong to other cellular organelles. Additionally, other lipids that were not analysed in the present study (e.g., cholesterol) are also regulators of membrane fluidity.

The attempt to evaluate significant changes in both high and low abundant PL molecular species led us to perform a univariate statistical analysis using autoscaled data. Importantly, whereas highly abundant PL species are central for maintaining structural and biophysical properties, lower abundant PL species might more likely act as mediators of signalling functions.

Treatment with H_2_O_2_ resulted in a decrease of two PC molecular species esterified to saturated fatty acids, namely PC (30:0) and PC (32:0), along with an increase of three (poly)-unsaturated PC molecular species, namely PC (34:1), PC (34:2) and PC (36:4) (Fig. 6 and Table 1). First, these results reflect the general increase of PL-DB1, PL-DB2, PL-DB3, and PL-DB4. More deeply, Cai and Harrison^26^ reviewed the regulation of EC by H_2_O_2_ and highlighted the fundamental role of this ROS in promoting the inflammatory state of the endothelium. In our conditions, treatment of BAEC with H_2_O_2_ led to the upregulation of PC (34:1), a ligand of the nuclear receptor PPARα^30^, that upon activation regulates several anti-inflammatory genes^31^. Thus, the up-regulation of PC (34:1) could be part of a protective strategy adopted by the cells in response to the inflammatory state promoted by oxidative stress.

We observed a significant up-regulation in the molecular species PE (34:3), PE (38:6), PE (36:3), PE (36:2) and PE (38:3) in BAEC treated with H_2_O_2_. PE is the second most abundant PL class in mammalian cells^32^, hence the increase of singular unsaturated molecular species correlates with the increase that we observed for PL-DB1, PL-DB2, PL-DB3, and PL-DB4. Due to the relatively small head group and the high unsaturation degree characterizing PE molecules, significant changes in the composition of PE species may affect the curvature of the cell membrane and contribute to an increase in its fluidity^33,34^ Of interest, Hailey and co-authors^35^ found mitochondrial PE to be the recruiters of autophagy markers in starved mammalian cells, while Rockenfeller and co-authors^36^ highlighted that increased levels of PE enhanced the lifespan of cultured mammalian cells via promotion of autophagy. Since high levels of H_2_O_2_ are known to induce apoptosis in cultured EC^26^, we postulate that the up-regulation of PE species herein observed is an adaptive mechanism of survival adopted by the cells in response to the early apoptotic signals triggered by the H_2_O_2_ treatment.

Curiously, H_2_O_2_ treatment led to a complex regulation of (poly)-unsaturated PS species in BAEC, since PS (38:4) and PS (40:7) were up-regulated, while PS (36:2) and PS (40:5) were down-regulated. The anti-inflammatory properties of PS in mammalian cells have recently been highlighted^37^, hence the increase of PS (38:4) and PS (40:7) could represent a mechanism promoted by BAEC to compensate the inflammatory state triggered by H_2_O_2_^26^. Since the formation of ox-PS in mammalian cell membranes is a hallmark of apoptosis^38,39^, we interpret that the decrease in (poly)-unsaturated PS is a consequence of the early apoptotic signals that might have been triggered by incubation with H_2_O_2_.

Five SM species were up-regulated after treatment with H_2_O_2_, namely SM (34:2), SM (36:2), SM (38:2) and SM (40:2). In eukaryotic cells, SM is known to co-localize and associate with sterol lipids and display structural roles related to cholesterol homeostasis and membrane distribution^40^. The interaction between SM and cholesterol forms ordered domains that are primarily involved in the regulation of cell membrane fluidity. In this sense, the oxidative stress-induced up-regulation of SM species observed in this study might reflect a mechanism of perturbation in the fluidity of the plasma membrane of treated BAEC. Nevertheless, there are other SM cellular functions that deserve discussion. Yang *et al*.^41^ treated yeast cells with increasing H_2_O_2_ concentrations and found that cell death effects were strongly abrogated in cells expressing sphingomyelin synthase 1 (SMS1), the principal enzyme catalysing the synthesis of cellular SM species. Since BAEC always maintained cell viability along the 24 h treatment with H_2_O_2_ (no cell death was observed), the significant increase in SM molecular species might represent an augmented SMS1 activity, adopted by the cells to abolish the cell death signals induced by H_2_O_2_.

Altogether, the significant variations herein observed for several PL and SM molecular species might arise from a wide array of BAEC adaptive mechanisms occurring upon H_2_O_2_-induced stress, which include response to inflammation, impaired membrane fluidity, and cell death. However, it is important to remark that none of the above-listed responses was measured in the present study, hence future works will be necessary to validate what has been herein conjectured.

The global observation of PL features highlighted that the treatment with glucose reduced the levels of PL-DB1, PL-DB2, PL-DB3, and PL-DB4 when compared with control (Fig. 5). A similar trend was observed for PL-C32, PL-C34, PL-C36, and PL-C38 (Fig. 5). To the best of our knowledge, the effects of hyperglycemia on the phospholipidome of EC have never been reported before. In this regard, it is interesting to pinpoint the experiments of Mīinea *et al*.^42^, which found a diminished Δ5 desaturase activity and a markedly decreased biosynthesis of arachidonic acid-containing PL in Schwann cells grown in 30 mM glucose.

The majority of the PL species that showed significant variation, reported in Fig. 6 and Table 1, were down-regulated after the treatment with glucose. The alterations induced by high glucose may reflect several mechanisms including biological signalling, redox imbalance and formation of advanced glycation end products (AGEs). Hempel and co-authors^43^ observed an increased EC permeability mediated by the activation of PKC, upon hyperglycemia. Duffy and co-authors reported that 72 h hyperglycemia induced apoptosis in human aortic EC (HAEC)^44^. Noteworthy, an excess of glucose can lead to NADPH consumption and formation of sorbitol, which in turn can induce oxidative and osmotic stress, respectively^45^. Several authors have reported the promotion of ROS generation and the redox imbalance mediated by high glucose in EC^46–48^. The ability of high glucose to induce 12/15-lipoxygenase (12/15-LOX) in mesangial cells^49^ and in EC^50^ was also highlighted. Glucose is also known to modify proteins^51^ and aminophospholipids (PE and PS)^52^. Altogether, these mechanisms are referred as glucotoxicity^53^ and contribute to the endothelial dysfunction that occurs in diabetes^54^. However, the cellular mechanisms linking glucotoxicity and PL turnover in mammalian cells are still unclear. On one hand, the formation of aminophospholipid-AGEs could have contributed to the decreased levels observed for PE and PS species; additionally, PE AGEs were found to trigger oxidative stress^55^, which can be a further explanation of the down-regulation of (poly)-unsaturated PL species observed in glucose-treated BAEC. On the other hand, the decrease of (poly)-unsaturated PL species may have been induced by the radical-based oxidative stress promoted by the 24 h exposition to high glucose. However, the ability of 12/15-LOX to oxidize (poly)-unsaturated fatty acid esterified to PL has recently been suggested^56^, hence a glucose-induced up-regulation of 12/15-LOX could be another explanation for the decrease in (poly)-unsaturated PL.

We analysed the global changes occurring in the PL features of BAEC after the treatment with HNE. The levels of PL-DB1, PL-DB2, and PL-DB3, along with the levels of PL-C32, PL-C34, PL-C36, and PL-C40 were downregulated when compared with controls (Fig. 5). Our work constitutes the first report on the effects of HNE on the phospholipidome of BAEC. The treatment with HNE also led to a down-regulation of all the PL molecular species reported in Fig. 6 and Table 1. HNE induces several intracellular cascades in EC, as reviewed by Chapple and co-authors^12^. Increased levels of this ALPP (1–100 µM) were found in disease states, mediating cellular-damaging pathways, including increased ROS generation^57^ and endoplasmic reticulum (ER) stress, altogether leading to EC dysfunction. Although the interplay between HNE-mediated toxicity (lipotoxicity) and the alteration of EC lipidome is mostly unknown at present, the ER stress induced by HNE in EC^58^ should be carefully considered, since this organelle is directly involved in the biosynthesis of several PL classes^32^. The down-regulation that we observed for all the PL species can be due to a HNE-induced perturbation of the PL biosynthetic pathways in the ER. However, BAEC treated with HNE were also found to suffer increased intracellular ROS levels, increased apoptosis and down-regulation of antioxidant defences^57^. This oxidative stress response triggered by HNE in BAEC can finally lead to membrane PL peroxidation, which would explain the decreased levels of (poly)-unsaturated PL species that were herein observed. Moreover, similarly to glucose, HNE is able to form covalent adducts with nucleophilic sites in aminophospholipids^59,60^, which could further explain the decrease of PS and PE molecular species in HNE-treated BAEC.

In summary, the results from the present study point out that the phospholipidome of BAEC suffers statistically significant changes upon different biochemical stresses (H_2_O_2_, glucose, and HNE). For the first time, the phopsholipidomic profiling of BAEC was compared between homeostasis, oxidative stress, glucotoxicity and lipotoxicity, and specific lipidome alterations were reported for all the tested conditions. The molecular adaptation observed for the treatment with H_2_O_2_ was distinctive when compared with the alterations induced by glucose or HNE. More deeply, we found H_2_O_2_ to increase the cellular levels of (poly)-unsaturated PL molecular species, while the same species were down-regulated after the treatment with glucose or HNE. These evidences highlight that PL are fundamental players in the response of vascular cells to such external stresses. A specific adaptation of the whole PL profile of EC can represent a cellular hallmark for the onset and the development of CVDs, atherosclerosis, and diabetes. Nevertheless, the biochemical mechanisms adopted by BAEC that lead to the alteration of the phospholipidome are still unclear, particularly in the case of glucolipotoxicity, and a considerable gap of knowledge exists regarding the lipid profiling of EC in non-homeostatic or pathological conditions. These results open new avenues of research towards further studies necessary to unveil the interplay between biochemical stress, PL turnover, and onset of cardiovascular pathologies and diabetic complications.

## Methods

### Reagents/chemicals

Phospholipid internal standards 1,2-dimyristoyl-*sn*-glycero-3-phosphocholine (dMPC), 1,2-dimyristoyl-*sn*-glycero-3-phosphoethanolamine (dMPE), 1,2-dimyristoyl-*sn*-glycero-3-phospho-(10-rac-)glycerol (dMPG), 1,2-dimyristoyl-*sn*-glycero-3-phospho-L-serine (dMPS), tetramyristoylcardiolipin (TMCL), 1,2-dipalmitoyl-*sn*-glycero-3-phosphatidylinositol (dPPI), N-palmitoyl-D-*erythro*-sphingosylphosphorylcholine (NPSM), 1-nonadecanoyl-2-hydroxy-*sn*-glycero-3-phosphocholine (LPC) were purchased from Avanti Polar Lipids, Inc. (Alabaster, AL). Chloroform, methanol and acetonitrile were purchased from Fisher scientific (Leicestershire, UK); all the solvents were of high performance liquid chromatography (HPLC) grade and were used without further purification. All the other reagents and chemicals used were of the highest grade of purity commercially available. The water was of Milli-Q purity (Synergy1, Millipore Corporation, Billerica, MA).

### Cell culture and treatments

Bovine aortic endothelial cells (BAEC) were obtained from Lonza, Inc., (Walkersville, MD) and cultured in RPMI1640 medium supplemented with antibiotics (100 U/mL penicillin and 100 µg/mL streptomycin) and 10% calf serum from Gibco (Life Technologies). For each experiment, BAEC were used between passages 8 and 16 and were grown to near confluence for experiments (80–90% density). Milli-Q water was used as a solvent throughout the experiments (H_2_O_2_, glucose, HNE). Treatment of BAEC with the different stressing agents (H_2_O_2_, glucose, HNE) was carried out in serum-free medium. This induces a near-quiescent state and does not reduce cell viability^61^. Control cells received an equivalent amount of water as required. Cells were subjected to biochemical stress with 1 mM H_2_O_2_, 25 mM glucose, or 10 µM HNE during 24 h. After treatment, cells were collected by scraping in PBS on ice and centrifuged at 1000 rpm for 5 min. Cell pellets were stored at −80 °C. The whole experimental procedure, including cell culture and treatments, lipid extraction and analysis, was repeated four times.

### Lipid extraction and quantification of phospholipid content

Thereafter, total lipids were extracted using the Bligh and Dyer method^62^, and the phospholipid (PL) amount in each lipid extract was quantified with the phosphorus assay, performed according to Bartlett and Lewis^63^. For the detailed experimental procedures of lipid extraction and PL quantification, the reader is referred to a previously published work in which the same methodologies were applied^64^.

### HPLC-ESI-MS and MS/MS analysis

Phospholipids were separated by hydrophilic interaction liquid chromatography (HILIC-LC-MS) using a high performance-LC (HPLC) system (Thermo scientific Accela^TM^) with an autosampler coupled online to the Q-Exactive® hybrid quadrupole Orbitrap® mass spectrometer (Thermo Fisher Scientific, Bremen, Germany). The solvent system consisted of two mobile phases as follows: mobile phase A [acetonitrile:methanol:water 50:25:25 (v/v/v) with 1 mM ammonium acetate] and mobile phase B [acetonitrile:methanol 60:40 (v/v) with 1 mM ammonium acetate]. Initially, 0% of mobile phase A was held isocratically for 8 min, followed by a linear increase to 60% of A within 7 min and a maintenance period of 15 min, returning to the initial conditions in 10 min. A volume of 5 µL of each sample containing 5 µg of phospholipid extract, 4 µL of phospholipid standards mix (dMPC - 0.02 µg, dMPE - 0.02 µg, NPSM - 0.02 µg, LPC - 0.02 µg, TMCL - 0.08 µg, dPPI - 0.08 µg, dMPG - 0.012 µg, dMPS - 0.04 µg) and 91 µL of eluent B was introduced into the Ascentis® Si column (15 cm × 1 mm, 3 µm, Sigma-Aldrich) with a flow rate of 40 µL min^−1^ and at 30 °C. The mass spectrometer with Orbitrap® technology operated simultaneously in positive (electrospray voltage 3.0 kV) and negative (electrospray voltage −2.7 kV) modes with a high resolution of 70,000 and AGC target 1e6. The capillary temperature was 250 °C and the sheath gas flow was 15 U. No intensity threshold was used during full MS acquisitions. PC and LPC species were analysed in the LC-MS spectra in the negative ion mode as acetate anions adducts [M + CH_3_COO]^−^. PE, PS, PI, PG and CL species were also analysed in the LC-MS spectra in the negative ion mode, as [M − H]^−^ ions. SM molecular species were analysed in the LC-MS spectra in positive ion mode as [M + H]^+^ ions. In MS/MS experiments, a resolution of 17,500 and AGC target of 1e5 were used and the cycles consisted of one full scan mass spectrum and ten data-dependent MS/MS scans that were repeated continuously throughout the experiments, with the dynamic exclusion of 60 seconds and intensity threshold of 1e4. Normalized collision energy™ (CE) ranged between 25, 30 and 35 eV. At least one blank run was performed between different treatment samples to prevent cross-contamination. Data acquisition was carried out using the Xcalibur data system (V3.3, Thermo Fisher Scientific, USA).

### Data and statistical analysis

Phospholipid peak integration and assignments were performed using MZmine 2.30^65^. The software allows for filtering and smoothing, peak detection, peak processing, and assignment against an in-house database. During the processing of the raw data acquired in full MS mode, all the peaks with raw intensity lower than 1e4 were excluded. For all assignments, peaks within 6 ppm of the lipid exact mass were considered. Consequently, assigned PL were further validated by manual analysis of the MS/MS data (Supplementary Table S1). Analysis of the MS/MS spectra acquired in positive ion mode was performed to confirm the identity of the molecular species belonging to the PC, LPC, SM, and PE classes. The fragment ion at *m/z* 184.07, when observed in the MS/MS acquired in the positive mode, indicates the presence of phosphocholine head group, allowing to identify the precursor ions as belonging to the PC, LPC and SM classes. The species belonging to these three classes were further differentiated by the characteristic retention times. The neutral loss of 141 Da, when observed in the MS/MS acquired in the positive mode, allows identifying precursor ions of the PE class. The MS/MS spectra acquired in negative ion mode were used to confirm the identity of PG, PI and PS phospholipids. In particular, the fragment ion at m/z 171.01 was used to confirm [M-H]^−^ PG class precursor ions and the fragment ion at *m/z* 241.01 was used to confirm [M − H]^−^ PI class precursor ions. The neutral loss of 87 Da, when observed in the MS/MS spectra acquired in the negative mode, allows identifying precursor ions belonging to the PS class. Negative ion mode MS/MS data were used to identify the fatty acid carboxylate anions fragments RCOO^−^, which allowed the assignment of the fatty acyl chains esterified to the PL precursor. All the MS/MS fragmentation patterns characteristic for the lipid classes analysed in the present study, acquired in positive and negative ion modes, are available online as supplementary information.

Relative quantitation was performed by exporting integrated peak areas values into a computer spreadsheet (Excel, Microsoft, Redmond, WA). For normalization of the data, the peak areas of the XICs of the PL precursors of each class (listed in Supplementary Table S1), (C:N composition), were divided for the peak area of the IS selected for the class.

Initial data assessment was performed using Metaboanalyst^66^. Shapiro-Wilk test was used for analysing the normality of the BAEC treatment data. Univariate statistical analysis was performed using ANOVA test following post hoc least significant difference (LSD) test, with Benjamin–Hochberg correction for the false discovery rate (FDR). Univariate data processing and analyses were performed using the SPSS software package (IBM SPSS Statistics Version 24). Principal component analysis (PCA), projection to latent structures discriminant analysis (PLS-DA) and hierarchical clustering were performed on auto-scaled data using the R software (R version 3.4.2 downloaded from https://www.R-project.org) with the packages RFmarkerDetector, FactoMineR^67^, Factoextra^68^ and Ropls^69^. PCA was performed using the FactoMineR and ellipses were drawn assuming a multivariate normal distribution. Hierarchical clustering was performed using Ward’s method using the method hclust. Boxplots were created using the R package ggplot^70^. Bar graphs were created using the software GraphPad Prism 7.

### Data availability

The datasets generated during and/or analysed during the current study are available online as supplementary material.

## Electronic supplementary material

Supplementary Information

Supplementary Table S1

Supplementary Table S2

## References

[1] Cines DB (1998). Endothelial cells in physiology and in the pathophysiology of vascular disorders. Blood.

[2] Cai H, Harrison DG (2000). Endothelial dysfunction in cardiovascular diseases: the role of oxidant stress. Circ. Res..

[3] Gimbrone MA, García-Cardeña G (2016). Endothelial cell dysfunction and the pathobiology of atherosclerosis. Circ. Res..

[4] Alexander RW (1995). Hypertension and the Pathogenesis of Atherosclerosis: Oxidative Stress and the Mediation of Arterial Inflammatory Response: A New Perspective. Hypertension.

[5] Heitzer T, Schlinzig T, Krohn K, Meinertz T, Münzel T (2001). Endothelial dysfunction, oxidative stress, and risk of cardiovascular events in patients with coronary artery disease. Circulation.

[6] Wang, L. *et al*. High glucose induces and activates Toll-like receptor 4 in endothelial cells of diabetic retinopathy. *Diabetol*. *Metab*. *Syndr*. **7** (2015). 10.1186/s13098-015-0086-4 PMC4604707 26468333

[7] Roy S, Ha J, Trudeau K, Beglova E (2010). Vascular Basement Membrane Thickening in Diabetic Retinopathy. Curr. Eye Res..

[8] van den Oever IAM, Raterman HG, Nurmohamed MT, Simsek S (2010). Endothelial Dysfunction, Inflammation, and Apoptosis in Diabetes Mellitus. Mediators Inflamm..

[9] Nakagami H, Kaneda Y, Ogihara T, Morishita R (2005). Endothelial dysfunction in hyperglycemia as a trigger of atherosclerosis. Curr. Diabetes Rev..

[10] Hou Q, Lei M, Hu K, Wang M (2015). The Effects of High Glucose Levels on Reactive Oxygen Species-Induced Apoptosis and Involved Signaling in Human Vascular Endothelial Cells. Cardiovasc. Toxicol..

[11] Herbst U, Toborek M, Kaiser S, Mattson MP, Hennig B (1999). 4-Hydroxynonenal induces dysfunction and apoptosis of cultured endothelial cells. J. Cell. Physiol..

[12] Chapple SJ, Cheng X, Mann GE (2013). Effects of 4-hydroxynonenal on vascular endothelial and smooth muscle cell redox signaling and function in health and disease. Redox Biol..

[13] Riahi Y (2015). Foam cell-derived 4-hydroxynonenal induces endothelial cell senescence in a TXNIP-dependent manner. J. Cell. Mol. Med..

[14] Gargiulo S (2017). Oxysterols and 4-hydroxy-2-nonenal contribute to atherosclerotic plaque destabilization. Free Radic. Biol. Med..

[15] Savage DB, Petersen KF, Shulman GI (2007). Disordered Lipid Metabolism and the Pathogenesis of Insulin Resistance. Physiol. Rev..

[16] Lim WLF, Martins IJ, Martins RN (2014). The Involvement of Lipids in Alzheimer’s Disease. J. Genet. Genomics.

[17] Linton, M. F. *et al*. The Role of Lipids and Lipoproteins in Atherosclerosis. In *Endotext* (eds De Groot, L. J. *et al*.) (MDText.com, Inc., 2000). 26844337

[18] Watson AD (2006). *Thematic review series: Systems Biology Approaches to Metabolic and Cardiovascular Disorders*. Lipidomics: a global approach to lipid analysis in biological systems. J. Lipid Res..

[19] Meikle PJ, Wong G, Barlow CK, Kingwell BA (2014). Lipidomics: Potential role in risk prediction and therapeutic monitoring for diabetes and cardiovascular disease. Pharmacol. Ther..

[20] Murphy EJ, Joseph L, Stephens R, Horrocks LA (1992). Phospholipid Composition of Cultured Human Endothelial Cells. Lipids.

[21] Héliès-Toussaint C (2006). Lipid metabolism in human endothelial cells. Biochim. Biophys. Acta BBA - Mol. Cell Biol. Lipids.

[22] Stegemann, C. *et al*. Comparative lipidomics profiling of human atherosclerotic plaques. *Circ*. *Cardiovasc*. *Genet*. CIRCGENETICS–110 (2011). 10.1161/CIRCGENETICS.110.959098 21511877

[23] Kolovou, G., Kolovou, V. & Mavrogeni, S. Lipidomics in vascular health: current perspectives. *Vasc*. *Health Risk Manag*., 333, 10.2147/VHRM.S54874 (2015). 10.2147/VHRM.S54874 PMC4472029 26109865

[24] Yang J, Yang S, Gao X, Yuan Y-J (2011). Integrative investigation of lipidome and signal pathways in human endothelial cells under oxidative stress. Mol. Biosyst..

[25] Klose C (2012). Flexibility of a Eukaryotic Lipidome – Insights from Yeast Lipidomics. Plos One.

[26] Cai H (2005). Hydrogen peroxide regulation of endothelial function: Origins, mechanisms, and consequences. Cardiovasc. Res..

[27] Peterson B, Stovall K, Monian P, Franklin JL, Cummings BS (2008). Alterations in phospholipid and fatty acid lipid profiles in primary neocortical cells during oxidant-induced cell injury. Chem. Biol. Interact..

[28] Cevc G (1991). How membrane chain-melting phase-transition temperature is affected by the lipid chain asymmetry and degree of unsaturation: an effective chain-length model. Biochemistry (Mosc.).

[29] Sergent O (2004). Role for Membrane Fluidity in Ethanol-Induced Oxidative Stress of Primary Rat Hepatocytes. J. Pharmacol. Exp. Ther..

[30] Chakravarthy MV (2009). Identification of a Physiologically Relevant Endogenous Ligand for PPARα in Liver. Cell.

[31] Straus DS, Glass CK (2007). Anti-inflammatory actions of PPAR ligands: new insights on cellular and molecular mechanisms. Trends Immunol..

[32] Vance JE (2008). Thematic Review Series: Glycerolipids. Phosphatidylserine and phosphatidylethanolamine in mammalian cells: two metabolically related aminophospholipids. J. Lipid Res..

[33] Verkleij AJ, Leunissen-Bijvelt J, de Kruijff B, Hope M, Cullis PR (1984). Non-bilayer structures in membrane fusion. Ciba Found. Symp..

[34] Lee AG (2004). How lipids affect the activities of integral membrane proteins. Biochim. Biophys. Acta BBA - Biomembr..

[35] Hailey DW (2010). Mitochondria Supply Membranes for Autophagosome Biogenesis during Starvation. Cell.

[36] Rockenfeller P (2015). Phosphatidylethanolamine positively regulates autophagy and longevity. Cell Death Differ..

[37] Yeom M (2013). Phosphatidylserine inhibits inflammatory responses in interleukin-1β–stimulated fibroblast-like synoviocytes and alleviates carrageenan-induced arthritis in rat. Nutr. Res..

[38] Fabisiak JP, Tyurina YY, Tyurin VA, Lazo JS, Kagan VE (1998). Random versus selective membrane phospholipid oxidation in apoptosis: role of phosphatidylserine. Biochemistry (Mosc.).

[39] Greenberg ME (2006). Oxidized phosphatidylserine–CD36 interactions play an essential role in macrophage-dependent phagocytosis of apoptotic cells. J. Exp. Med..

[40] Slotte JP, Ramstedt B (2007). The functional role of sphingomyelin in cell membranes. Eur. J. Lipid Sci. Technol..

[41] Yang Z, Khoury C, Jean-Baptiste G, Greenwood MT (2006). Identification of mouse sphingomyelin synthase 1 as a suppressor of Bax-mediated cell death in yeast. FEMS Yeast Res..

[42] Mîinea C, Kuruvilla R, Merrikh H, Eichberg J (2002). Altered arachidonic acid biosynthesis and antioxidant protection mechanisms in Schwann cells grown in elevated glucose. J. Neurochem..

[43] Hempel A (1997). High Glucose Concentrations Increase Endothelial Cell Permeability via Activation of Protein Kinase C. Circ. Res..

[44] Duffy A (2006). Distinct Effects of High-Glucose Conditions on Endothelial Cells of Macrovascular and Microvascular Origins. Endothelium.

[45] Sánchez-Gómez, F. J. *et al*. Detoxifying Enzymes at the Cross-Roads of Inflammation, Oxidative Stress, and Drug Hypersensitivity: Role of Glutathione Transferase P1-1 and Aldose Reductase. *Front*. *Pharmacol*. **7** (2016). 10.3389/fphar.2016.00237 PMC4973429 27540362

[46] Sheu ML (2008). Inhibition of NADPH oxidase-related oxidative stress-triggered signaling by honokiol suppresses high glucose-induced human endothelial cell apoptosis. Free Radic. Biol. Med..

[47] Ho FM (2006). High glucose-induced apoptosis in human vascular endothelial cells is mediated through NF-κB and c-Jun NH2-terminal kinase pathway and prevented by PI3K/Akt/eNOS pathway. Cell. Signal..

[48] Aljofan, M. & Ding, H. High glucose increases expression of cyclooxygenase-2, increases oxidative stress and decreases the generation of nitric oxide in mouse microvessel endothelial cells. *J*. *Cell*. *Physiol*. n/a-n/a, 10.1002/jcp.21986 (2009). 10.1002/jcp.21986 19950211

[49] Kang S-W (2001). 12-Lipoxygenase is increased in glucose-stimulated mesangial cells and in experimental diabetic nephropathy. Kidney Int..

[50] Ibrahim AS (2015). A lipidomic screen of hyperglycemia-treated HRECs links 12/15-Lipoxygenase to microvascular dysfunction during diabetic retinopathy via NADPH oxidase. J. Lipid Res..

[51] Vistoli G (2013). Advanced glycoxidation and lipoxidation end products (AGEs and ALEs): an overview of their mechanisms of formation. Free Radic. Res..

[52] Fountain WC (1999). Quantification of N-(Glucitol)ethanolamine and N-(Carboxymethyl)serine: Two Products of Nonenzymatic Modification of Aminophospholipids Formed *in Vivo*. Anal. Biochem..

[53] An X (2016). Mesenchymal Stem Cells Ameliorated Glucolipotoxicity in HUVECs through TSG-6. Int. J. Mol. Sci..

[54] Avogaro A, Albiero M, Menegazzo L, de Kreutzenberg S, Fadini GP (2011). Endothelial Dysfunction in Diabetes: The role of reparatory mechanisms. Diabetes Care.

[55] Oak J-H, Nakagawa K, Miyazawa T (2000). Synthetically prepared Amadori-glycated phosphatidylethanolamine can trigger lipid peroxidation via free radical reactions. FEBS Lett..

[56] Hammond VJ (2012). Novel Keto-phospholipids Are Generated by Monocytes and Macrophages, Detected in Cystic Fibrosis, and Activate Peroxisome Proliferator-activated Receptor-. J. Biol. Chem..

[57] Whitsett J, Picklo MJ, Vasquez-Vivar J (2007). 4-Hydroxy-2-Nonenal Increases Superoxide Anion Radical in Endothelial Cells via Stimulated GTP Cyclohydrolase Proteasomal Degradation. Arterioscler. Thromb. Vasc. Biol..

[58] Vladykovskaya E (2012). Lipid Peroxidation Product 4-Hydroxy- *trans* -2-nonenal Causes Endothelial Activation by Inducing Endoplasmic Reticulum Stress. J. Biol. Chem..

[59] Bacot S (2003). Covalent binding of hydroxy-alkenals 4-HDDE, 4-HHE, and 4-HNE to ethanolamine phospholipid subclasses. J. Lipid Res..

[60] Guo L, Davies SS (2013). Bioactive aldehyde-modified phosphatidylethanolamines. Biochimie.

[61] Oeste CL, Seco E, Patton WF, Boya P, Pérez-Sala D (2013). Interactions between autophagic and endo-lysosomal markers in endothelial cells. Histochem. Cell Biol..

[62] Bligh EG, Dyer WJ (1959). A rapid method of total lipid extraction and purification. Can. J. Biochem. Physiol..

[63] Bartlett EM, Lewis DH (1970). Spectrophotometric determination of phosphate esters in the presence and absence of orthophosphate. Anal. Biochem..

[64] Sousa B (2016). Alteration in Phospholipidome Profile of Myoblast H9c2 Cell Line in a Model of Myocardium Starvation and Ischemia: Phospholipidome Profile of Myoblasts H9c2. J. Cell. Physiol..

[65] Pluskal T, Castillo S, Villar-Briones A, Oresic M (2010). MZmine 2: modular framework for processing, visualizing, and analyzing mass spectrometry-based molecular profile data. BMC Bioinformatics.

[66] Xia J, Sinelnikov IV, Han B, Wishart DS (2015). MetaboAnalyst 3.0—making metabolomics more meaningful. Nucleic Acids Res..

[67] Lê, S., Josse, J. & Husson, F. FactoMineR: An R Package for Multivariate Analysis. *J*. *Stat*. *Softw*. **25** (2008).

[68] Hegde, V. Dimensionality reduction technique for developing undergraduate student dropout model using principal component analysis through R package. In, 1–6, 10.1109/ICCIC.2016.7919670 (IEEE, 2016).

[69] Turkmen AS, Billor N (2013). Influence Function Analysis for the Robust Partial Least Squares (RoPLS) Estimator. Commun. Stat. - Theory Methods.

[70] Wickham, H. *ggplot2: elegant graphics for data analysis*. (Springer, 2016).

