# Supplementary material for: Phospholipidome of endothelial cells shows a different adaptation response upon oxidative, glycative and lipoxidative stress
Source: Sci Rep. 2018 Aug 17;8:12365. doi: 10.1038/s41598-018-30695-0 (PMC6097988; doi:10.1038/s41598-018-30695-0)
Supplement: Supplementary file 1 — Supplementary Information [file 41598_2018_30695_MOESM1_ESM.docx]

**Supplementary Information**

**Phospholipidome of endothelial cells shows a different adaptation response upon oxidative, glycative and lipoxidative stress.**

Simone Colombo^1^, Tânia Melo^1^, Marta Martínez-López^2^, M. Jesús Carrasco^2^, Pedro Domingues^1^, Dolores Pérez-Sala^2^ and M. Rosário Domingues^1*^

^1^Mass Spectrometry Centre, Department of Chemistry & QOPNA, University of Aveiro, Campus Universitário de Santiago, 3810-193 Aveiro, Portugal

^2^Department of Structural and Chemical Biology, Centro de Investigaciones Biológicas, CSIC, Ramiro, de Maeztu, 9, 28040 Madrid, Spain

Corresponding author: M. Rosário Domingues^1^

^
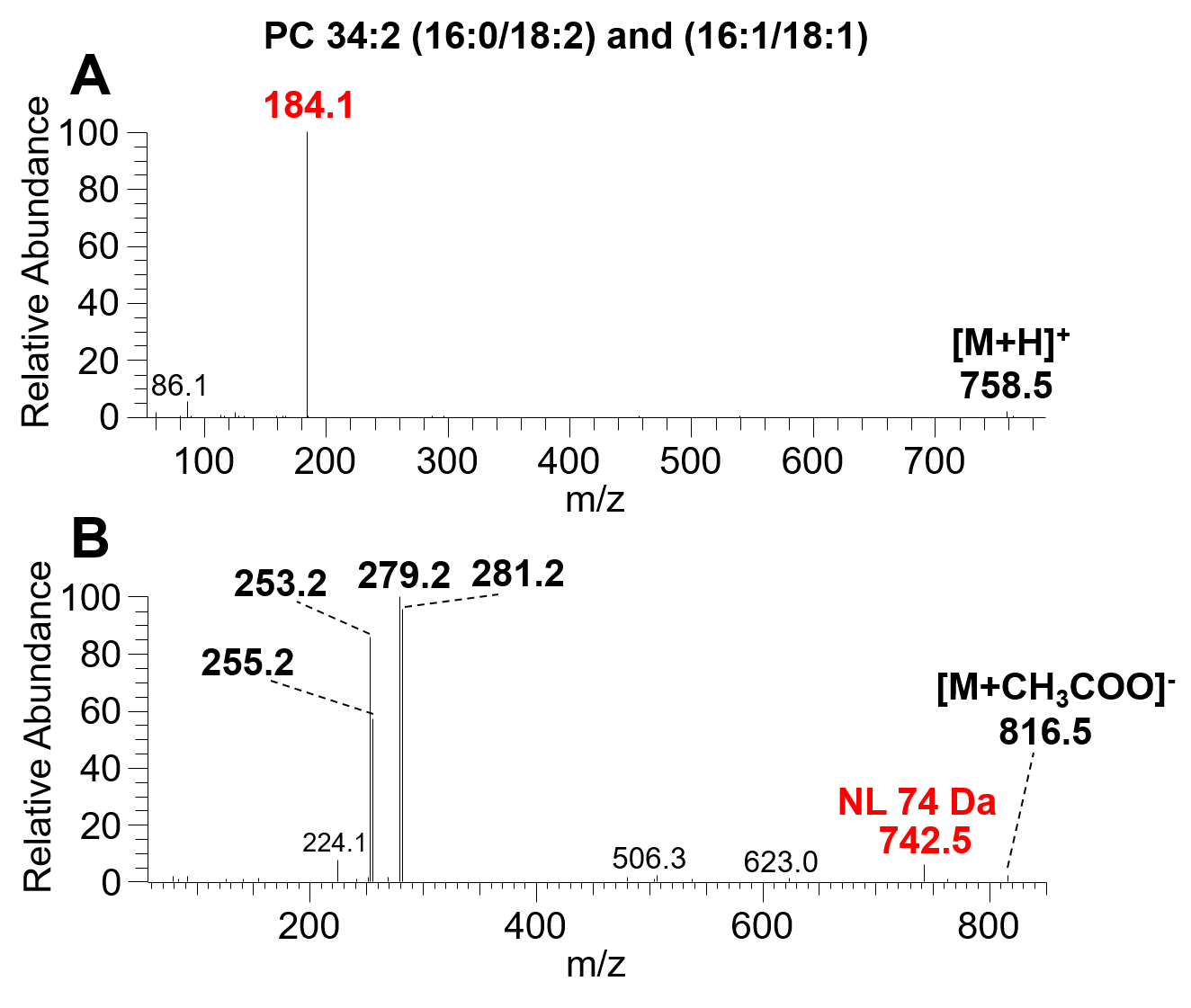
^

**Supplementary Figure S1**. A). ESI-MS/MS spectrum (HCD fragmentation) of the [M+H]^+^ ion of PC 34:2 (*m/z* 758.5). B) ESI-MS/MS spectrum (HCD fragmentation) of the [M+CH_3_COO]^-^ ion of PC 34:2 (*m/z* 816.5). Fragment ions characteristic for the PC class were highlighted in red.


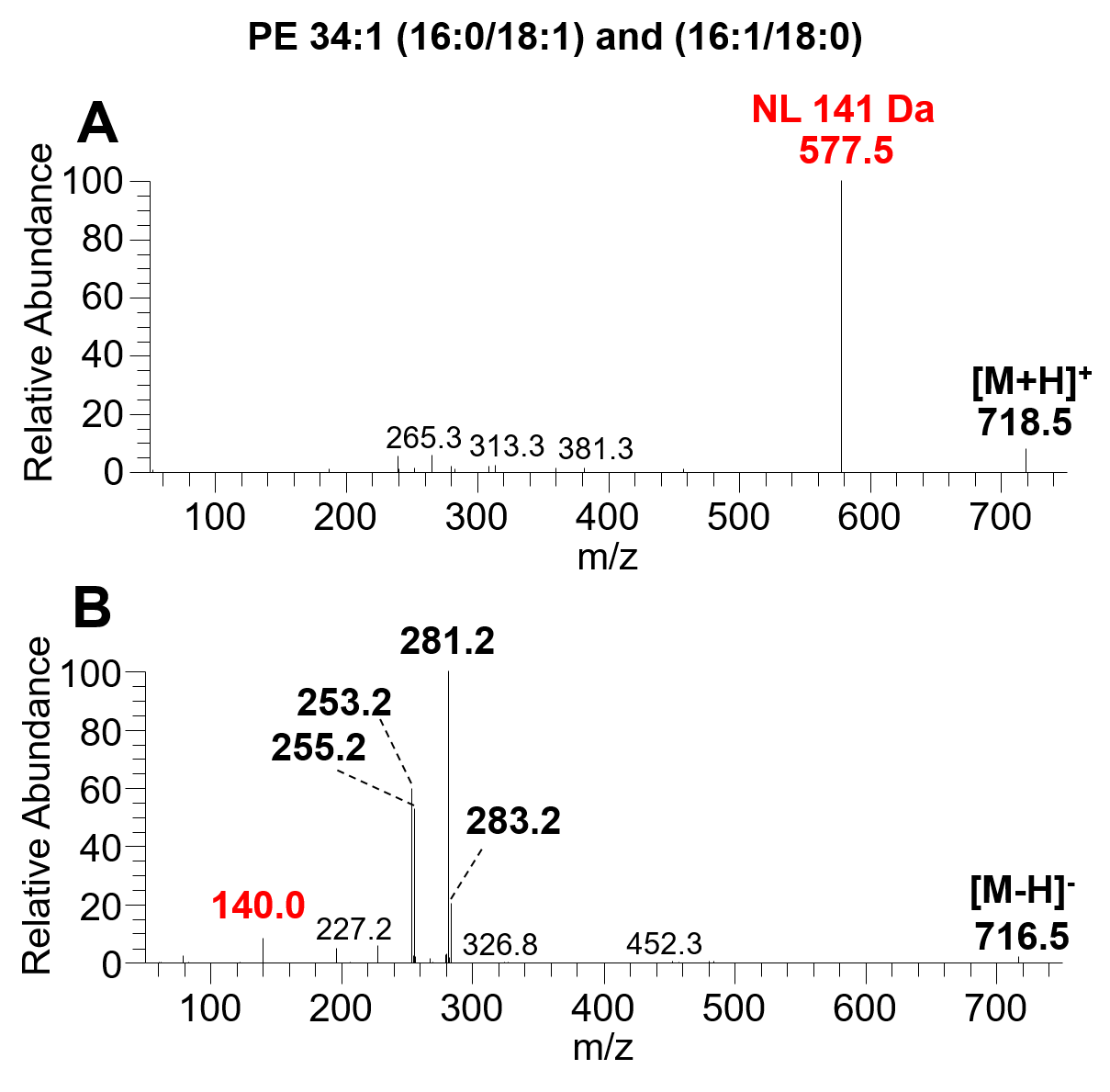


**Supplementary Figure S2**. A). ESI-MS/MS spectrum (HCD fragmentation) of the [M+H]^+^ ion of PE 34:1 (*m/z* 718.5). B) ESI-MS/MS spectrum (HCD fragmentation) of the [M-H]^-^ ion of PE 34:1 (*m/z* 716.5). Fragment ions characteristic for the PE class were highlighted in red.


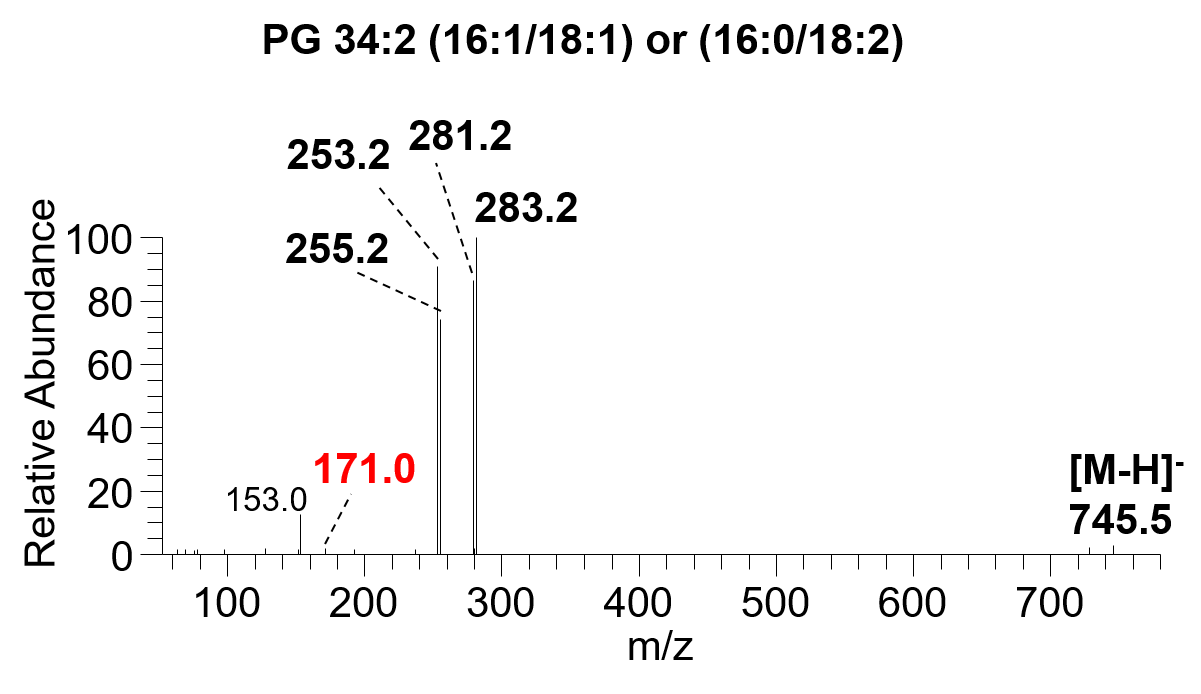


**Supplementary Figure S3**. ESI-MS/MS spectrum (HCD fragmentation) of the [M-H]^-^ ion of PG 34:2 (*m/z* 745.5). The fragment ion characteristic for the PG class was highlighted in red.


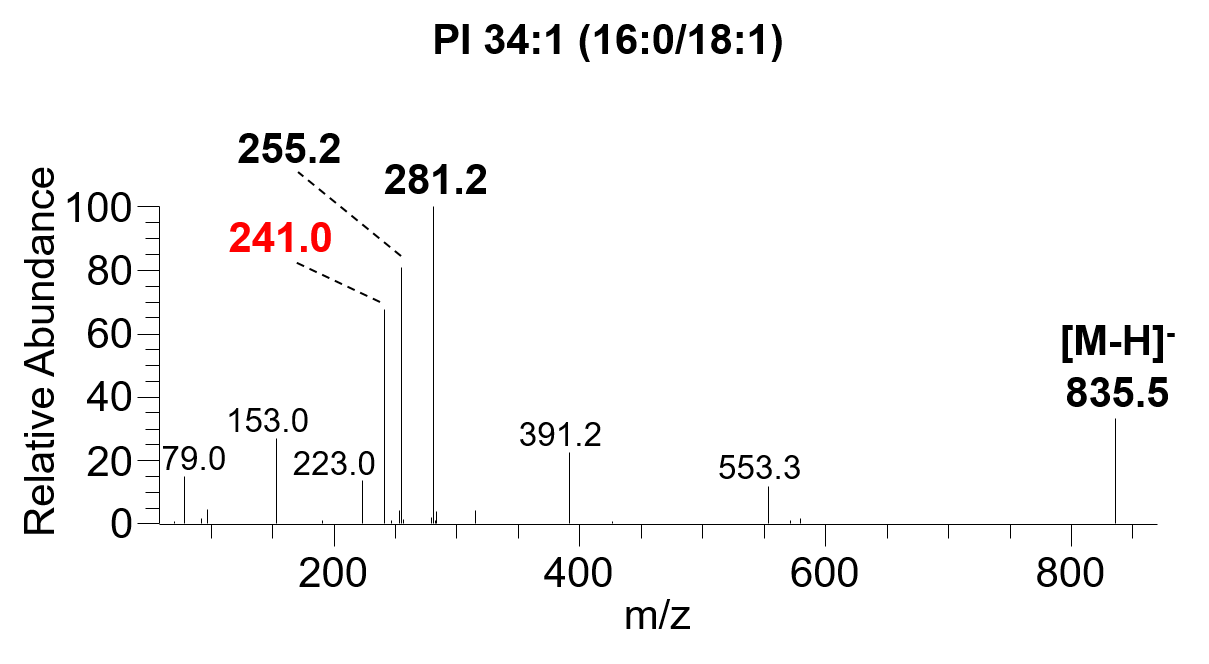


**Supplementary Figure S4**. ESI-MS/MS spectrum (HCD fragmentation) of the [M-H]^-^ ion of PI 34:1 (*m/z* 835.5). The fragment ion characteristic for the PI class was highlighted in red.


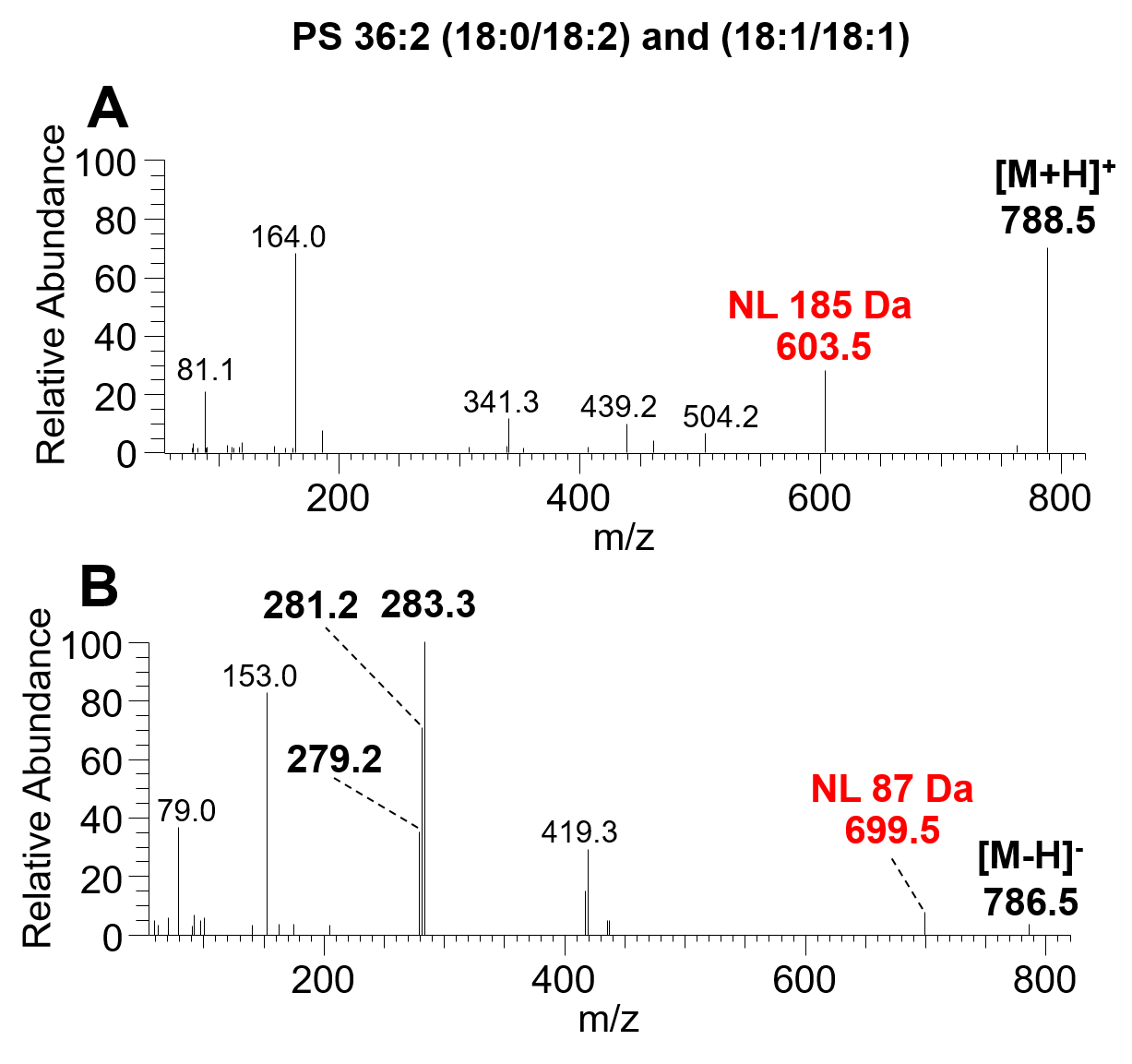


**Supplementary Figure S5**. A). ESI-MS/MS spectrum (HCD fragmentation) of the [M+H]^+^ ion of PS 36:2 (*m/z* 788.5). B) ESI-MS/MS spectrum (HCD fragmentation of the [M-H]^-^ ion of PS 36:2 (*m/z* 786.5). Fragment ions characteristic for the PS class were highlighted in red.


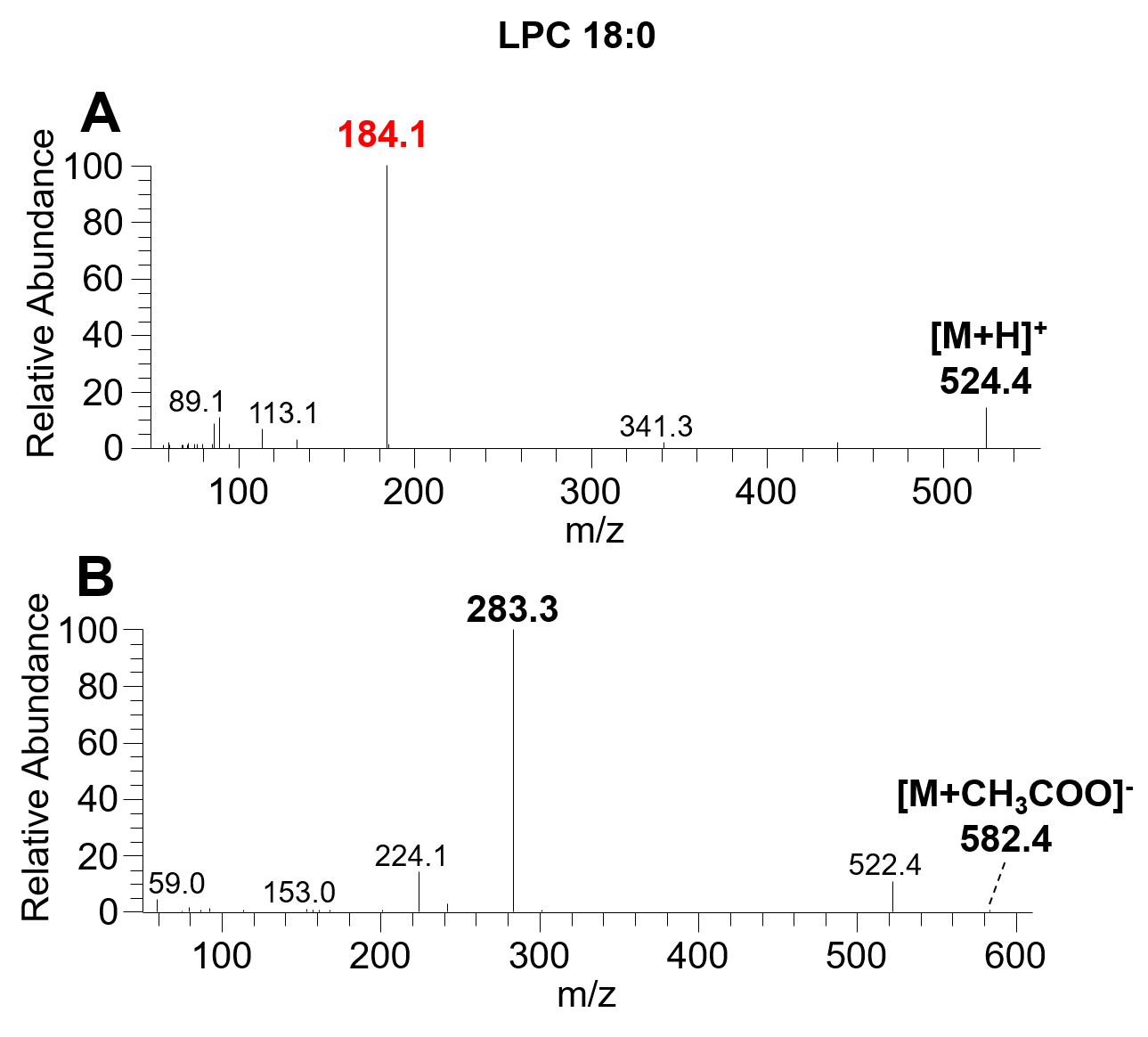


**Supplementary Figure S6**. A). ESI-MS/MS spectrum (HCD fragmentation) of the [M+H]^+^ ion of LPC 18:0 (*m/z* 524.4). B) ESI-MS/MS spectrum (HCD fragmentation of the [M+CH_3_COO]^-^ ion of LPC 18:0 (*m/z* 582.4). Fragment ions characteristic for the LPC class were highlighted in red.


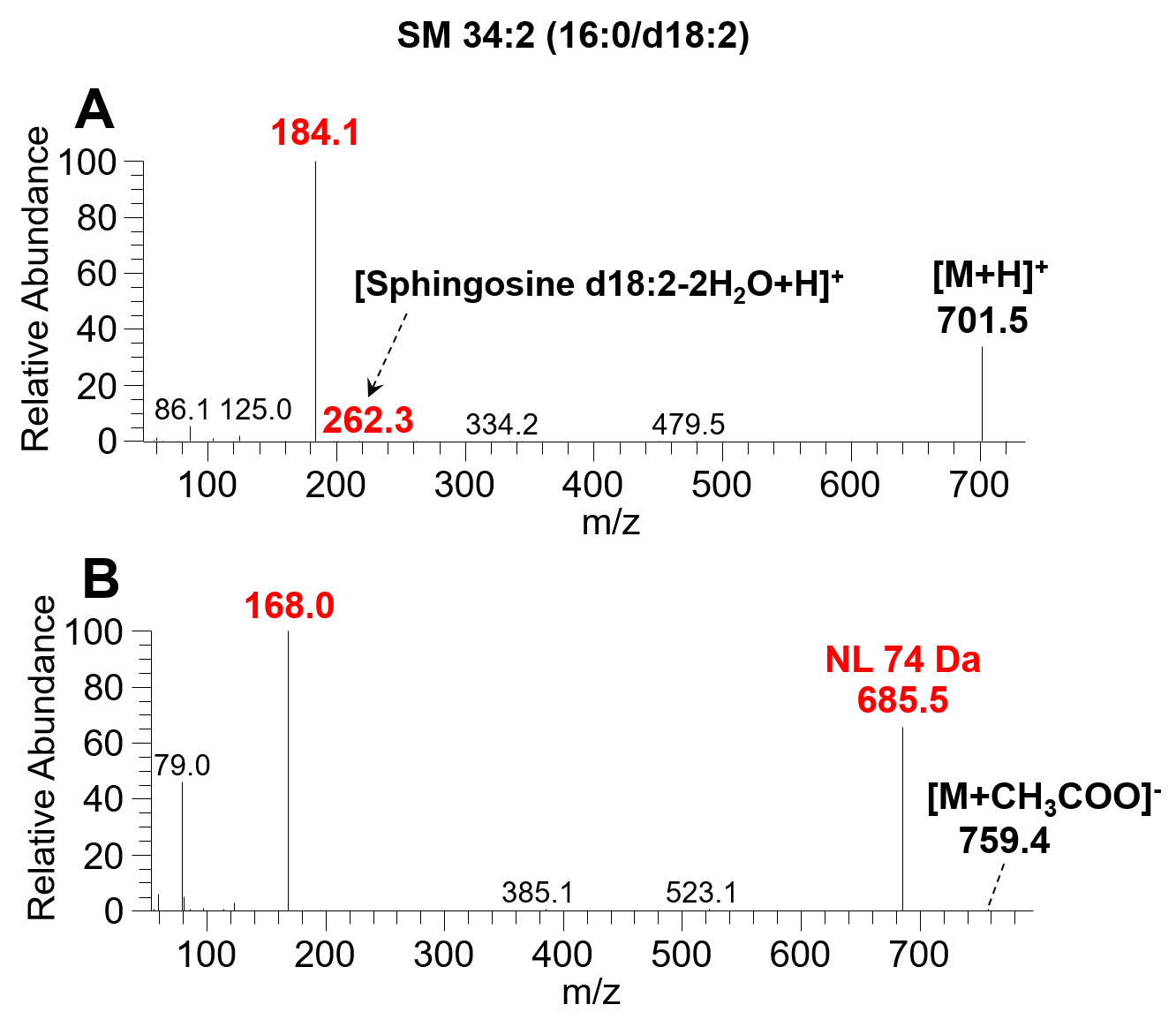


**Supplementary Figure S7** A). ESI-MS/MS spectrum (HCD fragmentation) of the [M+H]^+^ ion of SM 34:2 (*m/z* 701.5). B) ESI-MS/MS spectrum (HCD fragmentation) of the [M+CH_3_COO]^-^ ion of SM 34:2 (*m/z* 759.4). Fragment ions characteristic for the SM class were highlighted in red.


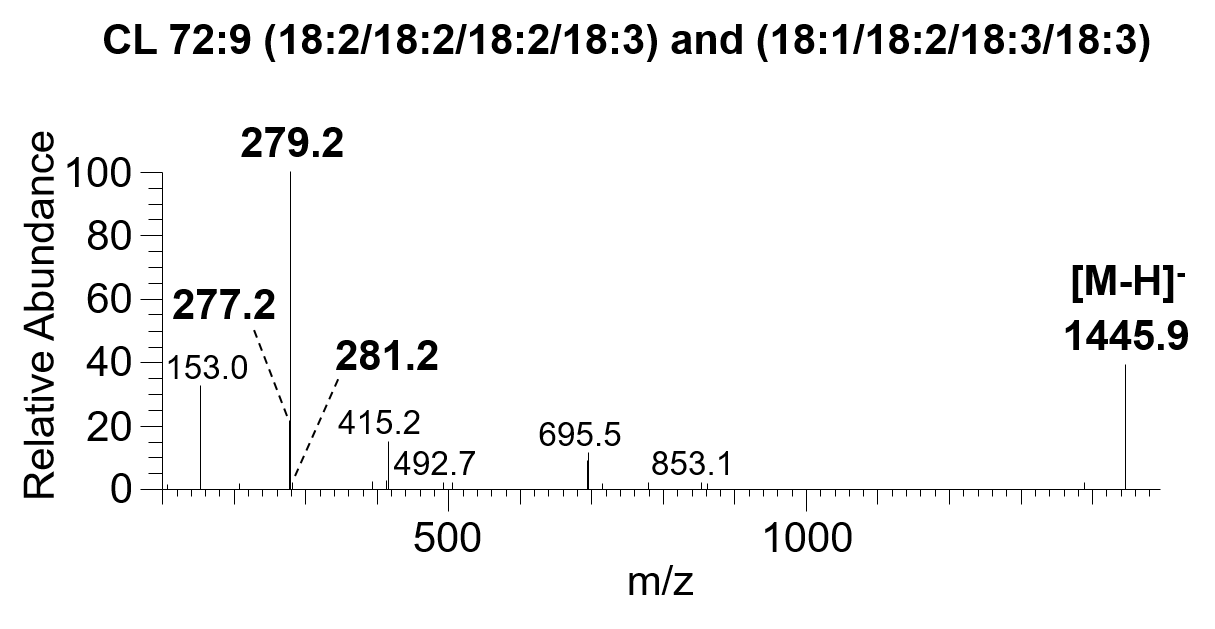
 **Supplementary Figure S8**. ESI-MS/MS spectrum (HCD fragmentation) of the [M-H]^-^ ion of CL 72:9 (*m/z* 1445.9).
